# Supplementary material for: Separation of Bioproducts through the Integration of Cyanobacterial Metabolism and Membrane Filtration: Facilitating Cyanobacteria’s Industrial Application
Source: Membranes (Basel). 2022 Sep 30;12(10):963. doi: 10.3390/membranes12100963 (PMC9611232; doi:10.3390/membranes12100963)
Supplement: Supplementary file 1 [file membranes-12-00963-s001.zip › membranes-1887230-supplementary.pdf]

Supplementary Information

Separation of Bioproducts through the Integration of Cyanobacterial Metabolism and Membrane Filtration: Facilitating Cyanobacteria's Industrial Application

Fei Hao [1,2,†], Xinyi Li [1,†], Jiameng Wang [3], Ruoyue Li [1], Liyan Zou [1], Kai Wang [1], Fuqing Chen [1], Feixiong Shi [1], Hui Yang [1,2,*,‡], Wen Wang [1,*,‡] and Miao Tian [1,*,‡]

1 School of Ecology and Environment, Northwestern Polytechnical University, Xi'an 710072, China
2 Center of Special Environmental Biomechanics & Biomedical Engineering, School of Life Sciences, Northwestern Polytechnical University, Xi'an 710072, China
3 School of Astronautics, Northwestern Polytechnical University, Xi'an 710072, China
* Correspondence: mailto: kittyyh@nwpu.edu.cn (H.Y.); wenwang@nwpu.edu.cn (W.W.); mtian@nwpu.edu.cn (M.T.)
† These authors contributed equally to this work.
‡ These authors jointly supervised this work.

Table S1. Oligonucleotides used in this study.

Name	Sequence (5'-3')
pcya-1-11	CACCGGAAGGAGCTGACTGGTATCAAGATTGCTGGGGAAGAAC
pcya-1-12	GATGCCCTTGAGAGCCTTCAACCTTCTGGAGCAGGAAGATGTC
pcya-1-13	CGGAAATGGCTTACGAACGGGAAGGCAGCGCTACCCAAATCG
pcya-1-14	CATCTTCCAGGAAATCTCCGCCGGGCATGGCAATGTCTCTCTG
pcya-1-15	GTTGAAGGCTCTCAAGGGCATCGGTCG
pcya-1-16	CCCGTTCGTAAGCCATTTCCGCTCGCC
pcya-1-17	GCGGAGATTTCCTGGAAGATGCCAGGAAG
pcya-1-18	CCAGTCAGCTCCTTCCGGTGGGC
pcya-2-21	CGCTCTCCCTTATGCGACTCCTGCATTAGGGGTGCCCATTGCTATCAGTTGTAAGT
pcya-2-22	ATGTTCAGAGCCATGGTGAAGGGCTCCTTCTGCAGGGTTCTCCTCGCTC
pcya-2-23	GCGAGGAGAACCCTGCAGAAGGAGCCCTTCACCATGGCTCTGAACAT
pcya-2-24	CTCAAATGCCTGAGGTTTCAGGCTCGGTACCCCTAGAGTC

pcya-2-25	CTGAAACCTCAGGCATTTGAGAAGCACACGG
pcya-2-26	CCTAATGCAGGAGTCGCATAAGGG

Table S2. Strains and plasmids used in this study.

**Strains**	**Genotype**	**References**
*Syn*7942	*Synechococcus elongatus* PCC 7942	Ducat et al.[1]
*Syn*6803	*Synechocystis* sp. PCC 6803	Wei et al.[2]
*Ana*7120	*Anabaena* sp. PCC 7120	Curatti et al.[3]
*Lep*0902	*Leptolyngbya* sp. Strain BL0902	Park et al.[4]
Tcya-1	*Syn*7942 + P_{psaAB}:: *cscB*; NSI targeting; cm^R	This study
**Plasmids**		
pcya-1	NSI targeting vector; P_{T7}; cm^R	This study
pcya-2	P_{psaAB}:: *cscB*; NSI targeting; cm^R	This study

Table S3. Template sequences of genes and plasmids used in this study.

Name	Sequence (5’-3’)
cscB	ATGGCTCTGAACATCCCCTTCCGCAACGCTTACTACCGCTTCGCTAGCAGCTACAGCTTCCTGTTCTTCATCAGCTGGAGCCTGTGGTGGAGC<br>CTGTACGCTATCTGGCTGAAAGGCCACCTGGGCCTGACCGGCACCGAACTGGGCACCCTGTACAGCGTGAACCAATTCACCAGCATCCTGTT<br>CATGATGTTCTACGGCATCGTGCAAGACAAACTGGGCCTGAAAAAACCCCTGATCTGGTGCATGAGCTTCATCCTGGTGCTGACCGGCCCCT<br>TCATGATCTACGTGTACGAACCCCTGCTGCAAAGCAACTTCAGCGTGGGCCTGATCCTGGGCGCTCTGTTCTTCGGCCTGGGCTACCTGGCTG<br>GCTGCGGCCTGCTGGACAGCTTCACCGAAAAAATGGCTCGCAACTTCCACTTCGAATACGGCACCGCTCGCGCTTGGGGCAGCTTCGGCTAT<br>GCTATAGGCGCGTTCTTCGCTGGCATATTCTTCAGCATCAGCCCCCACATCAACTTCTGGCTGGTGAGCCTGTTCGGCGCTGTGTTCATGATGA<br>TCAACATGCGCTTCAAAGACAAAGACCACCAATGCATCGCTGCTGACGCTGGCGGCGTGAAAAAAGAAGACTTCATCGCTGTGTTCAAAGA<br>CCGCAACTTCTGGGTGTTCGTGATCTTCATCGTGGGCACCTGGAGCTTCTACAACATCTTCGACCAACAACTGTTCCCCGTGTTCTACGCTGG<br>CCTGTTCGAAAGCCACGACGTGGGCACCCGCCTGTACGGCTACCTGAACAGCTTCCAAGTGGTGCTGGAAGCTCTGTGCATGGCTATCATCC<br>CCTTCTTCGTGAACCGCGTGGGCCCCAAAAACGCTCTGCTGATCGGCGTGGTGATCATGGCTCTGCGCATCCTGAGCTGCGCTCTGTTCGTGA<br>ACCCCTGGATCATCAGCCTGGTGAAACTGCTGCACGCTATCGAAGTGCCCCTGTGCGTGATCAGCGTGTTCAAATACAGCGTGGCTAACTTCG<br>ACAAACGCCTGAGCAGCACCATCTTCCTGATCGGCTTCCAAATCGCTAGCAGCCTGGGCATCGTGCTGCTGAGCACCCCCACCGGCATCCTG<br>TTCGACCACGCTGGCTACCAAACCGTGTTCTTCGCTATCAGCGGCATCGTGTGCCTGATGCTGCTGTTCGGCATCTTCTTCCTGAGCAAAAAA<br>CGCGAACAAATCGTGATGGAAACCCCCGTGCCCAGCGCTATCCACCACCACCACCACCACTAA
PpsaAB	GTGTTACGGCATTTTACTGACGGGTTAAGTAATCTTTAACAAAGATTTATGAGCCGTTACCGTAATTGCCCCCACAGGGGAACGCGATGTCTGT<br>GGACTCGCCCAGGACGTAATCAATTTTTCTGTACCGATATTAGCGGTGAAAAGTTTTATTCAACGTACTAAAATGCCCCGGCGGGAATTAACTT<br>GGGTTCCGGGAAGTCGGGTGCATTAGCCGTACTAGACTAACCCAATAGTTACTTTGTTTGATTCTTGATTTTGGAGACCGCTGATTTTA
NSI site	TATCAAGATTGCTGGGGAAGAACCGACCATCCACAACGCGATCGAGCGGCTGCTTGGCAAAAACCGTAAGGAAATCGAGCAAATTGCCAAG<br>GAGACCCTCGAAGGCAACTTGCGTGGTGTTTTAGCCAGCCTCACGCCGGAGCAGATCAACGAGGACAAAATTGCCTTTGCCAAAAGTCTGC<br>TGGAAGAGGCGGAGGATGACCTTGAGCAGCTGGGTCTAGTCCTCGATACGCTGCAAGTCCAGAACATTTCCGATGAGGTCGGTTATCTCTCG<br>GCTAGTGGACGCAAGCAGCGGGCTGATCTGCAGCGAGATGCCCGAATTGCTGAAGCCGATGCCCAGGCTGCCTCTGCGATCCAAACGGCCG<br>AAAATGACAAGATCACGGCCCTGCGTCGGATCGATCGCGATGTAGCGATCGCCCAAGCCGAGGCCGAGCGCCGGATTCAGGATGCGTTGAC<br>GCGGCGCGAAGCGGTGGTGGCCGAAGCTGAAGCGGACATTGCTACCGAAGTCGCTCGTAGCCAAGCAGAACTCCCTGTGCAGCAGGAGCG<br>GATCAAACAGGTGCAGCAGCAACTTCAAGCCGATGTGATCGCCCCAGCTGAGGCAGCTTGTAAACGGGCGATCGCGGAAGCGCGGGGGGC<br>CGCCGCCCGTATCGTCGAAGATGGAAAAGCTCAAGCGGAAGGGACCCAACGGCTGGCGGAGGCTTGGCAGACCGCTGGTGCTAATGCCCGC<br>GACATCTTCCTGCTCCAGAAGCTCGAGTCCCTGCTCGTCACGCTTTCAGGCACCGTGCCAGATATCGACGTGGAGTCGATCACTGTGATTGGC<br>GAAGGGGAAGGCAGCGCTACCCAAATCGCTAGCTTGCTGGAGAAGCTGAAACAAACCACGGGCATTGATCTGGCGAAATCCCTACCGGGTC<br>AATCCGACTCGCCCGCTGCGAAGTCCTAAGAGATAGCGATGTGACCGCGATCGCTTGTCAAGAATCCCAGTGATCCCGAACCATAGGAAGGC<br>AAGCTCAATGCTTGCCTCGTCTTGAGGACTATCTAGATGTCTGTGGAACGCACATTTATTGCCATCAAGCCCGATGGCGTTCAGCGGGGTTTG<br>GTCGGTACGATCATCGGCCGCTTTGAGCAAAAAGGCTTCAAACTGGTGGGCCTAAAGCAGCTGAAGCCCAGTCGCGAGCTGGCCGAACAGC<br>ACTATGCTGTCCACCGCGAGCGCCCCTTCTTCAATGGCCTCGTCGAGTTCATCACCTCTGGGCCGATCGTGGCGATCGTCTTGGAAGGCGAAG<br>GCGTTGTGGCGGCTGCTCGCAAGTTGATCGGCGCTACCAATCCGCTGACGGCAGAACCGGGCACCATCCGTGGTGATTTTGGTGTCAATATTG<br>GCCGCAACATCATCCATGGCTCGGATGCAATCGAAACAGCACAACAGGAAATTGCTCTCTGGTTTAGCCCAGCAGAGCTAAGTGATTGGACC<br>CCCACGATTCAACCCTGGCTGTACGAATAAGGTCTGCATTCCTTCAGAGAGACATTGCCATGCC
pcya-1	GGGGAATTGTGAGCGGATAACAATTCCCCTGTAGAAATAATTTTGTTTAACTTTAATAAGGAGATATACCATGGGCAGCAGCCATCACCATCAT<br>CACCACAGCCAGGATCCGAATTCGAGCTCGGCGCGCCTGCAGGTCGACAAGCTTGCGGCCGCATAATGCTTAAGTCGAACAGAAAGTAATC<br>GTATTGTACACGGCCGCATAATCGAAATTAATACGACTCACTATAGGGGAATTGTGAGCGGATAACAATTCCCCATCTTAGTATATTAGTTAAGT<br>ATAAGAAGGAGATATACATATGGCAGATCTCAATTGGATATCGGCCGGCCACGCGATCGCTGACGTCGGTACCCTCGAGTCTGGTAAAGAAAC<br>CGCTGCTGCGAAATTTGAACGCCAGCACATGGACTCGTCTACTAGCGCAGCTTAATTAACCTAGGCTGCTGCCACCGCTGAGCAATAACTAGC<br>ATAACCCCTTGGGGCCTCTAAACGGGTCTTGAGGGGTTTTTTGCTGAAACCTCAGGCATTTGAGAAGCACACGGTCACACTGCTTCCGGTAG<br>TCAATAAACCGGTAAACCAGCAATAGACATAAGCGGCTATTTAACGACCCTGCCCTGAACCGACGACCGGGTCGAATTTGCTTTCGAATTTCT

	GCCATTCATCCGCTTATTATCACTTATTCAGGCGTAGCACCAGGCGTTTAAGGGCACCAATAACTGCCTTAAAAAAATTACGCCCCGCCCTGCC<br>ACTCATCGCAGTACTGTTGTAATTCATTAAGCATTCTGCCGACATGGAAGCCATCACAGACGGCATGATGAACCTGAATCGCCAGCGGCATCA<br>GCACCTTGTCGCCTTGCGTATAATATTTGCCCATAGTGAAAACGGGGGCGAAGAAGTTGTCCATATTGGCCACGTTTAAATCAAAACTGGTGA<br>AACTCACCCAGGGATTGGCTGAGACGAAAAACATATTCTCAATAAACCCTTTAGGGAAATAGGCCAGGTTTTCACCGTAACACGCCACATCTT<br>GCGAATATATGTGTAGAAACTGCCGGAAATCGTCGTGGTATTCACTCCAGAGCGATGAAAACGTTTCAGTTTGCTCATGGAAAACGGTGTAAC<br>AAGGGTGAACACTATCCCATATCACCAGCTCACCGTCTTTCATTGCCATACGGAACTCCGGATGAGCATTCATCAGGCGGGCAAGAATGTGAA<br>TAAAGGCCGGATAAAACTTGTGCTTATTTTTCTTTACGGTCTTTAAAAAGGCCGTAATATCCAGCTGAACGGTCTGGTTATAGGTACATTGAGC<br>AACTGACTGAAATGCCTCAAAATGTTCTTTACGATGCCATTGGGATATATCAACGGTGGTATATCCAGTGATTTTTTTCTCCATTTTAGCTTCCTT<br>AGCTCCTGAAAATCTCGATAACTCAAAAAATACGCCCGGTAGTGATCTTATTTCATTATGGTGAAAGTTGGAACCTCTTACGTGCCGATCAACG<br>TCTCATTTTCGCCAAAAGTTGGCCCAGGGCTTCCCGGTATCAACAGGGACACCAGGATTTATTTATTCTGCGAAGTGATCTTCCGTCACAGGT<br>ATTTATTCGGCGCAAAGTGCGTCGGGTGATGCTGCCAACTTACTGATTTAGTGTATGATGGTGTTTTTGAGGTGCTCCAGTGGCTTCTGTTTCT<br>ATCAGCTGTCCCTCCTGTTCAGCTACTGACGGGGTGGTGCGTAACGGCAAAAGCACCGCCGGACATCAGCGCTAGCGGAGTGTATACTGGCT<br>TACTATGTTGGCACTGATGAGGGTGTCAGTGAAGTGCTTCATGTGGCAGGAGAAAAAAGGCTGCACCGGTGCGTCAGCAGAATATGTGATAC<br>AGGATATATTCCGCTTCCTCGCTCACTGACTCGCTACGCTCGGTCGTTCGACTGCGGCGAGCGGAAATGGCTTACGAACGGGAAGGCAGCGC<br>TACCCAAATCGCTAGCTTGCTGGAGAAGCTGAAACAAACCACGGGCATTGATCTGGCGAAATCCCTACCGGGTCAATCCGACTCGCCCGCTG<br>CGAAGTCCTAAGAGATAGCGATGTGACCGCGATCGCTTGTCAAGAATCCCAGTGATCCCGAACCATAGGAAGGCAAGCTCAATGCTTGCCTC<br>GTCTTGAGGACTATCTAGATGTCTGTGGAACGCACATTTATTGCCATCAAGCCCGATGGCGTTCAGCGGGGTTTGGTCGGTACGATCATCGGC<br>CGCTTTGAGCAAAAAGGCTTCAAACTGGTGGGCCTAAAGCAGCTGAAGCCCAGTCGCGAGCTGGCCGAACAGCACTATGCTGTCCACCGCG<br>AGCGCCCCTTCTTCAATGGCCTCGTCGAGTTCATCACCTCTGGGCCGATCGTGGCGATCGTCTTGGAAGGCGAAGGCGTTGTGGCGGCTGCT<br>CGCAAGTTGATCGGCGCTACCAATCCGCTGACGGCAGAACCGGGCACCATCCGTGGTGATTTTGGTGTCAATATTGGCCGCAACATCATCCAT<br>GGCTCGGATGCAATCGAAACAGCACAACAGGAAATTGCTCTCTGGTTTAGCCCAGCAGAGCTAAGTGATTGGACCCCCACGATTCAACCCTG<br>GCTGTACGAATAAGGTCTGCATTCCTTCAGAGAGACATTGCCATGCCCGGCGGAGATTTCCTGGAAGATGCCAGGAAGATACTTAACAGGGA<br>AGTGAGAGGGCCGCGGCAAAGCCGTTTTTCCATAGGCTCCGCCCCCCTGACAAGCATCACGAAATCTGACGCTCAAATCAGTGGTGGCGAA<br>ACCCGACAGGACTATAAAGATACCAGGCGTTTCCCCTGGCGGCTCCCTCGTGCGCTCTCCTGTTCCTGCCTTTCGGTTTACCGGTGTCATTCC<br>GCTGTTATGGCCGCGTTTGTCTCATTCCACGCCTGACACTCAGTTCCGGGTAGGCAGTTCGCTCCAAGCTGGACTGTATGCACGAACCCCCCG<br>TTCAGTCCGACCGCTGCGCCTTATCCGGTAACTATCGTCTTGAGTCCAACCCGGAAAGACATGCAAAAGCACCACTGGCAGCAGCCACTGGT<br>AATTGATTTAGAGGAGTTAGTCTTGAAGTCATGCGCCGGTTAAGGCTAAACTGAAAGGACAAGTTTTGGTGACTGCGCTCCTCCAAGCCAGT<br>TACCTCGGTTCAAAGAGTTGGTAGCTCAGAGAACCTTCGAAAAACCGCCCTGCAAGGCGGTTTTTTCGTTTTCAGAGCAAGAGATTACGCGC<br>AGACCAAAACGATCTCAAGAAGATCATCTTATTAATCAGATAAAATATTTCTAGATTTCAGTGCAATTTATCTCTTCAAATGTAGCACCTGAAGT<br>CAGCCCCATACGATATAAGTTGTAATTCTCATGTTAGTCATGCCCCGCGCCCACCGGAAGGAGCTGACTGGTATCAAGATTGCTGGGGAAGAA<br>CCGACCATCCACAACGCGATCGAGCGGCTGCTTGGCAAAAACCGTAAGGAAATCGAGCAAATTGCCAAGGAGACCCTCGAAGGCAACTTGC<br>GTGGTGTTTTAGCCAGCCTCACGCCGGAGCAGATCAACGAGGACAAAATTGCCTTTGCCAAAAGTCTGCTGGAAGAGGCGGAGGATGACCT<br>TGAGCAGCTGGGTCAAGTCCTCGATACGCTGCAAGTCCAGAACATTTCCGATGAGGTCGGTTATCTCTCGGCTAGTGGACGCAAGCAGCGGG<br>CTGATCTGCAGCGAGATGCCCGAATTGCTGAAGCCGATGCCCAGGCTGCCTCTGCGATCCAAACGGCCGAAAATGACAAGATCACGGCCCTG<br>CGTCGGATCGATCGCGATGTAGCGATCGCCCAAGCCGAGGCCGAGCGCCGGATTCAGGATGCGTTGACGCGGCGCGAAGCGGTGGTGGCCG<br>AAGCTGAAGCGGACATTGCTACCGAAGTCGCTCGTAGCCAAGCAGAACTCCCTGTGCAGCAGGAGCGGATCAAACAGGTGCAGCAGCAAC<br>TTCAAGCCGATGTGATCGCCCCAGCTGAGGCAGCTTGTAAACGGGCGATCGCGGAAGCGCGGGGGGCCGCCGCCCGTATCGTCGAAGATGG<br>AAAAGCTCAAGCGGAAGGGACCCAACGGCTGGCGGAGGCTTGGCAGACCGCTGGTGCTAATGCCCGCGACATCTTCCTGCTCCAGAAGGTT<br>GAAGGCTCTCAAGGGCATCGGTCGAGATCCCGGTGCCTAATGAGTGAGCTAACTTACATTAATTGCGTTGCGCTCACTGCCCGCTTTCCAGTC<br>GGGAAACCTGTCGTGCCAGCTGCATTAATGAATCGGCCAACGCGCGGGGAGAGGCGGTTTGCGTATTGGGCGCCAGGGTGGTTTTTCTTTTC<br>ACCAGTGAGACGGGCAACAGCTGATTGCCCTTCACCGCCTGGCCCTGAGAGAGTTGCAGCAAGCGGTCCACGCTGGTTTGCCCCAGCAGGC<br>GAAAATCCTGTTTGATGGTGGTTAACGGCGGGATATAACATGAGCTGTCTTCGGTATCGTCGTATCCCACTACCGAGATGTCCGCACCAACGC<br>GCAGCCCGGACTCGGTAATGGCGCGCATTGCGCCCAGCGCCATCTGATCGTTGGCAACCAGCATCGCAGTGGGAACGATGCCCTCATTCAGC<br>ATTTGCATGGTTTGTTGAAAACCGGACATGGCACTCCAGTCGCCTTCCCGTTCCGCTATCGGCTGAATTTGATTGCGAGTGAGATATTTATGCC

	AGCCAGCCAGACGCAGACGCGCCGAGACAGAACTTAATGGGCCCGCTAACAGCGCGATTTGCTGGTGACCCAATGCGACCAGATGCTCCAC<br>GCCCAGTCGCGTACCGTCTTCATGGGAGAAAATAATACTGTTGATGGGTGTCTGGTCAGAGACATCAAGAAATAACGCCGGAACATTAGTGC<br>AGGCAGCTTCCACAGCAATGGCATCCTGGTCATCCAGCGGATAGTTAATGATCAGCCCACTGACGCGTTGCGCGAGAAGATTGTGCACCGCC<br>GCTTTACAGGCTTCGACGCCGCTTCGTTCTACCATCGACACCACCACGCTGGCACCCAGTTGATCGGCGCGAGATTTAATCGCCGCGACAATT<br>TGCGACGGCGCGTGCAGGGCCAGACTGGAGGTGGCAACGCCAATCAGCAACGACTGTTTGCCCGCCAGTTGTTGTGCCACGCGGTTGGGAA<br>TGTAATTCAGCTCCGCCATCGCCGCTTCCACTTTTTCCCGCGTTTTCGCAGAAACGTGGCTGGCCTGGTTCACCACGCGGGAAACGGTCTGAT<br>AAGAGACACCGGCATACTCTGCGACATCGTATAACGTTACTGGTTTCACATTCACCACCCTGAATTGACTCTCTTCCGGGCGCTATCATGCCAT<br>ACCGCGAAAGGTTTTGCGCCATTCGATGGTGTCCGGGATCTCGACGCTCTCCCTTATGCGACTCCTGCATTAGGAAATTAATACGACTCACTAT<br>A
pcya-2	CTTATGCGACTCCTGCATTAGGGTGTTACGGCATTTTACTGACGGGTTAAGTAATCTTTAACAAAGATTTATGAGCCGTTACCGTAATTGCCCCC<br>ACAGGGGAACGCGATGTCTGTGGACTCGCCCAGGACGTAATCAATTTTTCTGTACCGATATTAGCGGTGAAAAGTTTTATTCAACGTACTAAA<br>ATGCCCCGGCGGGAATTAACTTGGGTTCCGGGAAGTCGGGTGCATTAGCCGTACTAGACTAACCCAATAGTTACTTTGTTTGATTCTTGATTTT<br>GGAGACCGCTGATTTTAgaaggagcccttcaccATGGCTCTGAACATCCCCTTCCGCAACGCTTACTACCGCTTCGCTAGCAGCTACAGCTTCCTGTTC<br>TTCATCAGCTGGAGCCTGTGGTGGAGCCTGTACGCTATCTGGCTGAAAGGCCACCTGGGCCTGACCGGCACCGAACTGGGCACCCTGTACAG<br>CGTGAACCAATTCACCAGCATCCTGTTCATGATGTTCTACGGCATCGTGCAAGACAAACTGGGCCTGAAAAAACCCCTGATCTGGTGCATGA<br>GCTTCATCCTGGTGCTGACCGGCCCCTTCATGATCTACGTGTACGAACCCCTGCTGCAAAGCAACTTCAGCGTGGGCCTGATCCTGGGCGCTC<br>TGTTCTTCGGCCTGGGCTACCTGGCTGGCTGCGGCCTGCTGGACAGCTTCACCGAAAAAATGGCTCGCAACTTCCACTTCGAATACGGCACC<br>GCTCGCGCTTGGGGCAGCTTCGGCTATGCTATAGGCGCGTTCTTCGCTGGCATATTCTTCAGCATCAGCCCCCACATCAACTTCTGGCTGGTGA<br>GCCTGTTCGGCGCTGTGTTCATGATGATCAACATGCGCTTCAAAGACAAAGACCACCAATGCATCGCTGCTGACGCTGGCGGCGTGAAAAAA<br>GAAGACTTCATCGCTGTGTTCAAAGACCGCAACTTCTGGGTGTTCGTGATCTTCATCGTGGGCACCTGGAGCTTCTACAACATCTTCGACCAA<br>CAACTGTTCCCCGTGTTCTACGCTGGCCTGTTCGAAAGCCACGACGTGGGCACCCGCCTGTACGGCTACCTGAACAGCTTCCAAGTGGTGCT<br>GGAAGCTCTGTGCATGGCTATCATCCCCTTCTTCGTGAACCGCGTGGGCCCCAAAAACGCTCTGCTGATCGGCGTGGTGATCATGGCTCTGCG<br>CATCCTGAGCTGCGCTCTGTTCGTGAACCCCTGGATCATCAGCCTGGTGAAACTGCTGCACGCTATCGAAGTGCCCCTGTGCGTGATCAGCGT<br>GTTCAAATACAGCGTGGCTAACTTCGACAAACGCCTGAGCAGCACCATCTTCCTGATCGGCTTCCAAATCGCTAGCAGCCTGGGCATCGTGCT<br>GCTGAGCACCCCCACCGGCATCCTGTTCGACCACGCTGGCTACCAAACCGTGTTCTTCGCTATCAGCGGCATCGTGTGCCTGATGCTGCTGTT<br>CGGCATCTTCTTCCTGAGCAAAAAACGCGAACAAATCGTGATGGAAACCCCCGTGCCCAGCGCTATCCACCACCACCACCACCACTAAccagctt<br>tcttgtacaaagtggtcctgcaggagaaggccatcctgacggatggccttttttgaagctgactctaggggtaccgagcCTGAAACCTCAGGCATTTGAGAAGCACACGGTCACACTGCTTC<br>CGGTAGTCAATAAACCGGTAAACCAGCAATAGACATAAGCGGCTATTTAACGACCCTGCCCTGAACCGACGACCGGGTCGAATTTGCTTTCG<br>AATTTCTGCCATTCATCCGCTTATTATCACTTATTCAGGCGTAGCACCAGGCGTTTAAGGGCACCAATAACTGCCTTAAAAAAATTACGCCCCG<br>CCCTGCCACTCATCGCAGTACTGTTGTAATTCATTAAGCATTCTGCCGACATGGAAGCCATCACAGACGGCATGATGAACCTGAATCGCCAGC<br>GGCATCAGCACCTTGTCGCCTTGCGTATAATATTTGCCCATAGTGAAAACGGGGGCGAAGAAGTTGTCCATATTGGCCACGTTTAAATCAAAA<br>CTGGTGAAACTCACCCAGGGATTGGCTGAGACGAAAAACATATTCTCAATAAACCCTTTAGGGAAATAGGCCAGGTTTTCACCGTAACACGC<br>CACATCTTGCGAATATATGTGTAGAAACTGCCGGAAATCGTCGTGGTATTCACTCCAGAGCGATGAAAACGTTTCAGTTTGCTCATGGAAAAC<br>GGTGTAACAAGGGTGAACACTATCCCATATCACCAGCTCACCGTCTTTCATTGCCATACGGAACTCCGGATGAGCATTCATCAGGCGGGCAAG<br>AATGTGAATAAAGGCCGGATAAAACTTGTGCTTATTTTTCTTTACGGTCTTTAAAAAGGCCGTAATATCCAGCTGAACGGTCTGGTTATAGGTA<br>CATTGAGCAACTGACTGAAATGCCTCAAAATGTTCTTTACGATGCCATTGGGATATATCAACGGTGGTATATCCAGTGATTTTTTTCTCCATTTT<br>AGCTTCCTTAGCTCCTGAAAATCTCGATAACTCAAAAAATACGCCCGGTAGTGATCTTATTTCATTATGGTGAAAGTTGGAACCTCTTACGTGC<br>CGATCAACGTCTCATTTTCGCCAAAAGTTGGCCCAGGGCTTCCCGGTATCAACAGGGACACCAGGATTTATTTATTCTGCGAAGTGATCTTCC<br>GTCACAGGTATTTATTCGGCGCAAAGTGCGTCGGGTGATGCTGCCAACTTACTGATTTAGTGTATGATGGTGTTTTTGAGGTGCTCCAGTGGCT<br>TCTGTTTCTATCAGCTGTCCCTCCTGTTCAGCTACTGACGGGGTGGTGCGTAACGGCAAAAGCACCGCCGGACATCAGCGCTAGCGGAGTGT<br>ATACTGGCTTACTATGTTGGCACTGATGAGGGTGTCAGTGAAGTGCTTCATGTGGCAGGAGAAAAAAGGCTGCACCGGTGCGTCAGCAGAAT<br>ATGTGATACAGGATATATTCCGCTTCCTCGCTCACTGACTCGCTACGCTCGGTCGTTCGACTGCGGCGAGCGGAAATGGCTTACGAACGGGAA<br>GGCAGCGCTACCCAAATCGCTAGCTTGCTGGAGAAGCTGAAACAAACCACGGGCATTGATCTGGCGAAATCCCTACCGGGTCAATCCGACTC<br>GCCCGCTGCGAAGTCCTAAGAGATAGCGATGTGACCGCGATCGCTTGTCAAGAATCCCAGTGATCCCGAACCATAGGAAGGCAAGCTCAATG

	CTTGCCTCGTCTTGAGGACTATCTAGATGTCTGTGGAACGCACATTTATTGCCATCAAGCCCGATGGCGTTCAGCGGGGTTTGGTCGGTACGAT<br>CATCGGCCGCTTTGAGCAAAAAGGCTTCAAACTGGTGGGCCTAAAGCAGCTGAAGCCCAGTCGCGAGCTGGCCGAACAGCACTATGCTGTC<br>CACCGCGAGCGCCCCTTCTTCAATGGCCTCGTCGAGTTCATCACCTCTGGGCCGATCGTGGCGATCGTCTTGGAAGGCGAAGGCGTTGTGGC<br>GGCTGCTCGCAAGTTGATCGGCGCTACCAATCCGCTGACGGCAGAACCGGGCACCATCCGTGGTGATTTTGGTGTCAATATTGGCCGCAACAT<br>CATCCATGGCTCGGATGCAATCGAAACAGCACAACAGGAAATTGCTCTCTGGTTTAGCCCAGCAGAGCTAAGTGATTGGACCCCCACGATTC<br>AACCCTGGCTGTACGAATAAGGTCTGCATTCCTTCAGAGAGACATTGCCATGCCCGGCGGAGATTTCCTGGAAGATGCCAGGAAGATACTTA<br>ACAGGGAAGTGAGAGGGCCGCGGCAAAGCCGTTTTTCCATAGGCTCCGCCCCCCTGACAAGCATCACGAAATCTGACGCTCAAATCAGTGG<br>TGGCGAAACCCGACAGGACTATAAAGATACCAGGCGTTTCCCCTGGCGGCTCCCTCGTGCGCTCTCCTGTTCCTGCCTTTCGGTTTACCGGTG<br>TCATTCCGCTGTTATGGCCGCGTTTGTCTCATTCCACGCCTGACACTCAGTTCCGGGTAGGCAGTTCGCTCCAAGCTGGACTGTATGCACGAA<br>CCCCCCGTTCAGTCCGACCGCTGCGCCTTATCCGGTAACTATCGTCTTGAGTCCAACCCGGAAAGACATGCAAAAGCACCACTGGCAGCAGC<br>CACTGGTAATTGATTTAGAGGAGTTAGTCTTGAAGTCATGCGCCGGTTAAGGCTAAACTGAAAGGACAAGTTTTGGTGACTGCGCTCCTCCA<br>AGCCAGTTACCTCGGTTCAAAGAGTTGGTAGCTCAGAGAACCTTCGAAAAACCGCCCTGCAAGGCGGTTTTTTCGTTTTCAGAGCAAGAGAT<br>TACGCGCAGACCAAAACGATCTCAAGAAGATCATCTTATTAATCAGATAAAATATTTCTAGATTTCAGTGCAATTTATCTCTTCAAATGTAGCAC<br>CTGAAGTCAGCCCCATACGATATAAGTTGTAATTCTCATGTTAGTCATGCCCCGCGCCCACCGGAAGGAGCTGACTGGTATCAAGATTGCTGG<br>GGAAGAACCGACCATCCACAACGCGATCGAGCGGCTGCTTGGCAAAAACCGTAAGGAAATCGAGCAAATTGCCAAGGAGACCCTCGAAGG<br>CAACTTGCGTGGTGTTTTAGCCAGCCTCACGCCGGAGCAGATCAACGAGGACAAAATTGCCTTTGCCAAAAGTCTGCTGGAAGAGGCGGAG<br>GATGACCTTGAGCAGCTGGGTCAAGTCCTCGATACGCTGCAAGTCCAGAACATTTCCGATGAGGTCGGTTATCTCTCGGCTAGTGGACGCAA<br>GCAGCGGGCTGATCTGCAGCGAGATGCCCGAATTGCTGAAGCCGATGCCCAGGCTGCCTCTGCGATCCAAACGGCCGAAAATGACAAGATC<br>ACGGCCCTGCGTCGGATCGATCGCGATGTAGCGATCGCCCAAGCCGAGGCCGAGCGCCGGATTCAGGATGCGTTGACGCGGCGCGAAGCGG<br>TGGTGGCCGAAGCTGAAGCGGACATTGCTACCGAAGTCGCTCGTAGCCAAGCAGAACTCCCTGTGCAGCAGGAGCGGATCAAACAGGTGCA<br>GCAGCAACTTCAAGCCGATGTGATCGCCCCAGCTGAGGCAGCTTGTAAACGGGCGATCGCGGAAGCGCGGGGGGCCGCCGCCCGTATCGTC<br>GAAGATGGAAAAGCTCAAGCGGAAGGGACCCAACGGCTGGCGGAGGCTTGGCAGACCGCTGGTGCTAATGCCCGCGACATCTTCCTGCTCC<br>AGAAGGTTGAAGGCTCTCAAGGGCATCGGTCGAGATCCCGGTGCCTAATGAGTGAGCTAACTTACATTAATTGCGTTGCGCTCACTGCCCGCT<br>TTCCAGTCGGGAAACCTGTCGTGCCAGCTGCATTAATGAATCGGCCAACGCGCGGGGAGAGGCGGTTTGCGTATTGGGCGCCAGGGTGGTTT<br>TTCTTTTCACCAGTGAGACGGGCAACAGCTGATTGCCCTTCACCGCCTGGCCCTGAGAGAGTTGCAGCAAGCGGTCCACGCTGGTTTGCCCC<br>AGCAGGCGAAAATCCTGTTTGATGGTGGTTAACGGCGGGATATAACATGAGCTGTCTTCGGTATCGTCGTATCCCACTACCGAGATGTCCGCA<br>CCAACGCGCAGCCCGGACTCGGTAATGGCGCGCATTGCGCCCAGCGCCATCTGATCGTTGGCAACCAGCATCGCAGTGGGAACGATGCCCTC<br>ATTCAGCATTTGCATGGTTTGTTGAAAACCGGACATGGCACTCCAGTCGCCTTCCCGTTCCGCTATCGGCTGAATTTGATTGCGAGTGAGATAT<br>TTATGCCAGCCAGCCAGACGCAGACGCGCCGAGACAGAACTTAATGGGCCCGCTAACAGCGCGATTTGCTGGTGACCCAATGCGACCAGAT<br>GCTCCACGCCCAGTCGCGTACCGTCTTCATGGGAGAAAATAATACTGTTGATGGGTGTCTGGTCAGAGACATCAAGAAATAACGCCGGAACA<br>TTAGTGCAGGCAGCTTCCACAGCAATGGCATCCTGGTCATCCAGCGGATAGTTAATGATCAGCCCACTGACGCGTTGCGCGAGAAGATTGTG<br>CACCGCCGCTTTACAGGCTTCGACGCCGCTTCGTTCTACCATCGACACCACCACGCTGGCACCCAGTTGATCGGCGCGAGATTTAATCGCCGC<br>GACAATTTGCGACGGCGCGTGCAGGGCCAGACTGGAGGTGGCAACGCCAATCAGCAACGACTGTTTGCCCGCCAGTTGTTGTGCCACGCGG<br>TTGGGAATGTAATTCAGCTCCGCCATCGCCGCTTCCACTTTTTCCCGCGTTTTCGCAGAAACGTGGCTGGCCTGGTTCACCACGCGGGAAAC<br>GGTCTGATAAGAGACACCGGCATACTCTGCGACATCGTATAACGTTACTGGTTTCACATTCACCACCCTGAATTGACTCTCTTCCGGGCGCTAT<br>CATGCCATACCGCGAAAGGTTTTGCGCCATTCGATGGTGTCCGGGATCTCGACGCTCTCC

Table S4. The characterization of MF and UF membranes.

	**MF010**	**UF050**
**Contact angle (°)**	**69.2±1.7**	**82.5±1.3**
**Mean pore size (nm)**	**95.4**	**16.6**

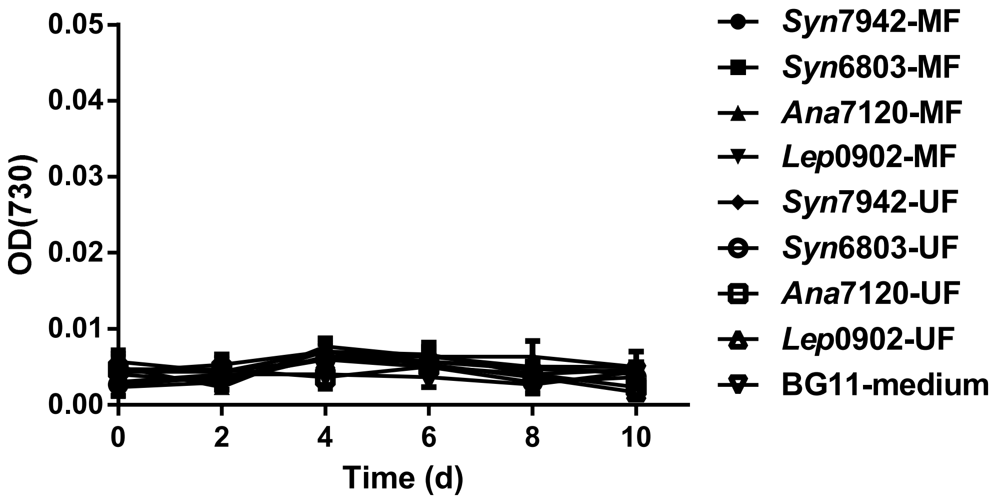

Figure S1. The detection of cyanobacteria existing in permeate solution after MF or UF filtration.

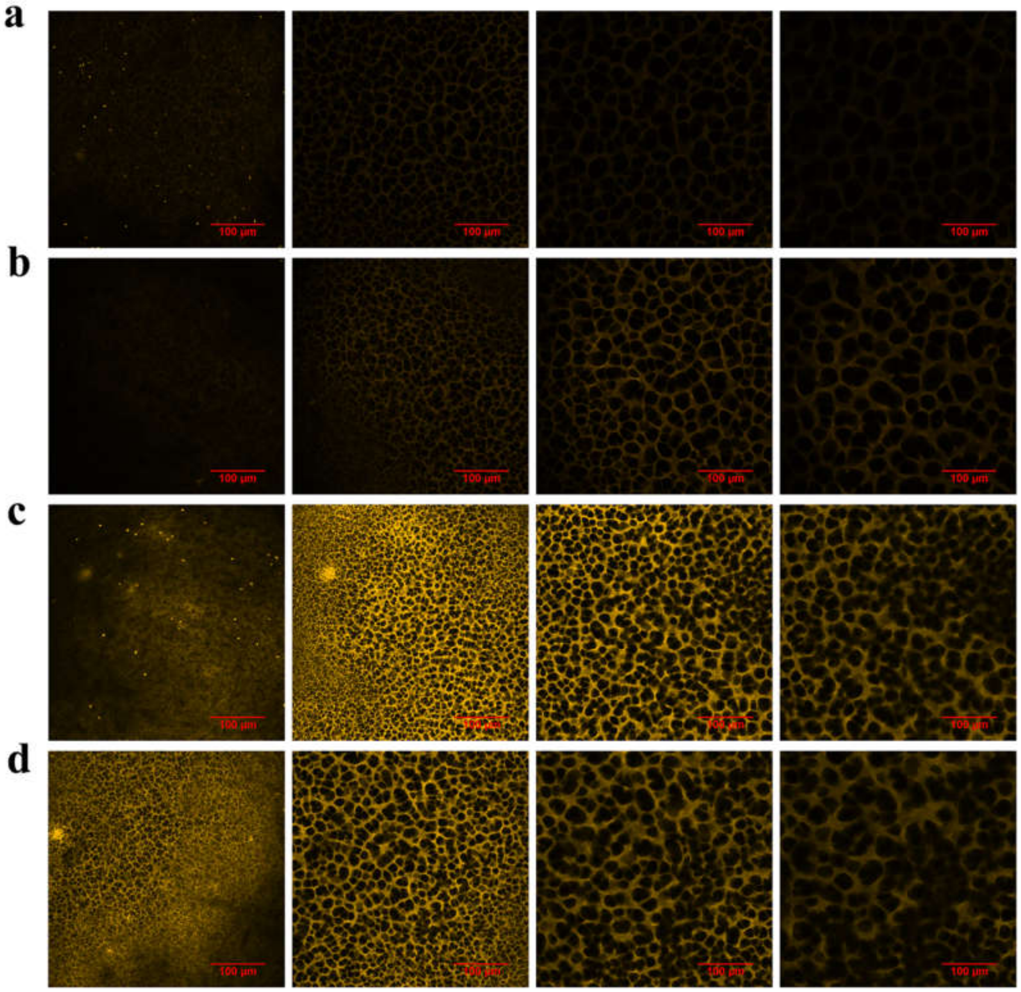

Figure S2. CLSM images of membranes after filtration of cyanobacteria. (**a-b**) Images (from left to right) of the surface of MF membranes after they were used to filter Syn7942 cells under a feed pressure of 0.34 bar (**a**) or 1.00 bar (**b**), (**c–d**) Images (from left to right) of the surface of UF membranes after they were used to filter Syn7942 cells under a feed pressure of 0.34 bar (**c**) or 1.00 bar (**d**). The imaging layers (from left to right) are 5-35 um depth from the surface of the MF or UF membrane.

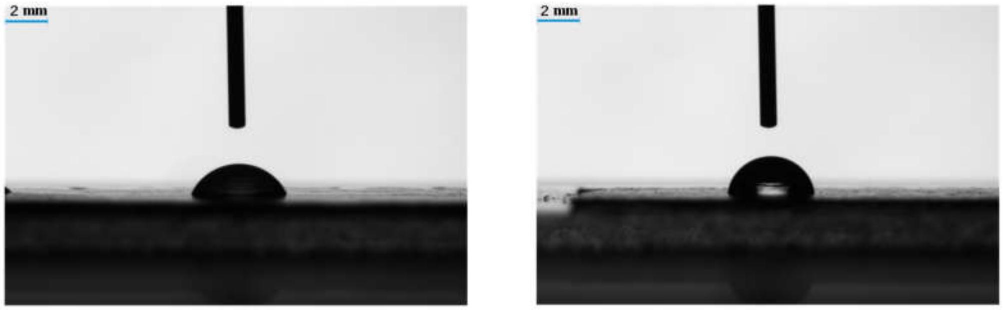

Figure S3. The contact angle of MF and UF membranes.

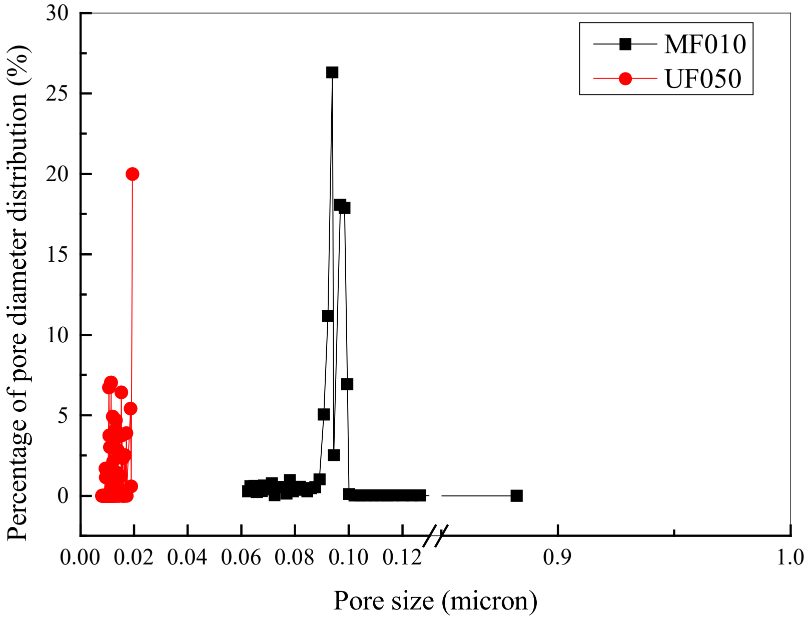

Figure S4. The pore size of MF and UF membranes.

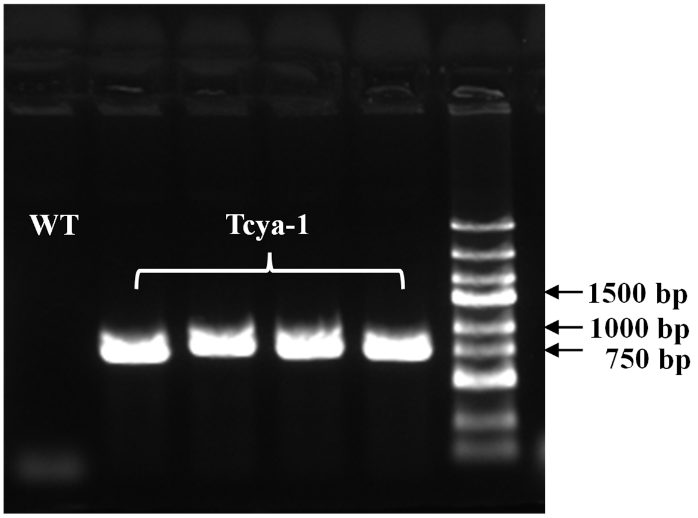

Figure S5. Colony PCR with gene- specific primers. The target size of PCR product is 904 bp (including *PpsaAB* sequences and part of *cscB* sequences). WT present wild type of *Syn*7942, and Tcya-1 present *cscB*$^{+}$ *Syn*7942.

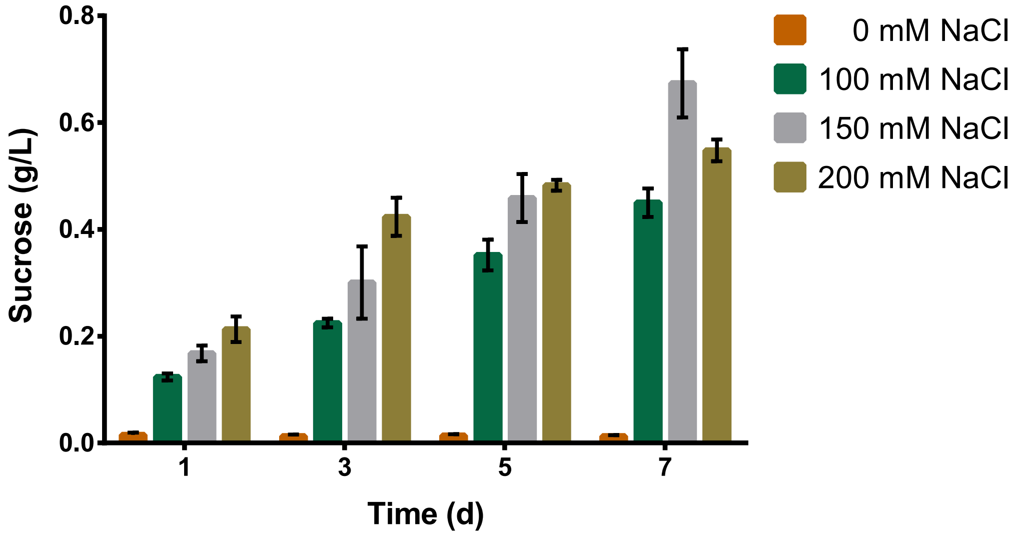

Figure S6. The secreted sucrose yield of Tcya-1 under different concentrations of NaCl shock. Error bars represent standard deviations (in triplicate).

References:

1. Ducat, D.C., Avelar-Rivas, J.A., Way, J.C. & Silver, P.A. Rerouting carbon flux to enhance photosynthetic productivity. *Appl Environ Microbiol* **78**, 2660-8 (2012).
2. Du, W., Liang, F., Duan, Y., Tan, X. & Lu, X. Exploring the photosynthetic production capacity of sucrose by cyanobacteria. *Metab Eng* **19**, 17-25 (2013).
3. Curatti, L., Giarrocco, L. & Salerno, G.L. Sucrose synthase and RuBisCo expression is similarly regulated by the nitrogen source in the nitrogen-fixing cyanobacterium Anabaena sp. *Planta* **223**, 891-900 (2006).
4. Park, S. *et al.* Gene Transfer in Leptolyngbya sp. Strain BL0902, a Cyanobacterium Suitable for Production of Biomass and Bioproducts. *PLoS ONE* **7**, e30901 (2012).
